# Cystic Echinococcosis in the Province of Álava, North Spain: The Monetary Burden of a Disease No Longer under Surveillance

**DOI:** 10.1371/journal.pntd.0003069

**Published:** 2014-08-07

**Authors:** Hélène Carabin, Francisco J. Balsera-Rodríguez, José Rebollar-Sáenz, Christine T. Benner, Aitziber Benito, Juan C. Fernández-Crespo, David Carmena

**Affiliations:** 1 Department of Biostatistics and Epidemiology, College of Public Health, University of Oklahoma Health Sciences Center, Oklahoma City, Oklahoma, United States of America; 2 Department of General Surgery, Section of Hepato-Biliopancreatic Surgery, Txagorritxu Hospital, Vitoria-Gasteiz, Spain; 3 Health Promotion Section, Department of Health, Basque Government, Vitoria-Gasteiz, Spain; 4 Laboratory of Public Health of Álava, Department of Health, Basque Government, Vitoria-Gasteiz, Spain; 5 Parasitology Service, National Centre for Microbiology, Health Institute Carlos III, Majadahonda, Madrid, Spain; University of Zurich, Switzerland

## Abstract

Cystic echinococcosis (CE) is endemic in Spain but has been considered non-endemic in the province of Álava, Northern Spain, since 1997. However, Álava is surrounded by autonomous regions with some of the highest CE prevalence proportions in the nation, casting doubts about the current classification. The purpose of this study is to estimate the frequency of CE in humans and animals and to use this data to determine the societal cost incurred due to CE in the Álava population in 2005. We have identified epidemiological and clinical data from surveillance and hospital records, prevalence data in intermediate (sheep and cattle) host species from abattoir records, and economical data from national and regional official institutions. Direct costs (diagnosis, treatment, medical care in humans and condemnation of offal in livestock species) and indirect costs (productivity losses in humans and reduction in growth, fecundity and milk production in livestock) were modelled using the Latin hypercube method under five different scenarios reflecting different assumptions regarding the prevalence of asymptomatic cases and associated productivity losses in humans. A total of 13 human CE cases were reported in 2005. The median total cost (95% credible interval) of CE in humans and animals in Álava in 2005 was estimated to range between €61,864 (95%CI%: €47,304–€76,590) and €360,466 (95%CI: €76,424–€752,469), with human-associated losses ranging from 57% to 93% of the total losses, depending on the scenario used. Our data provide evidence that CE is still very well present in Álava and incurs important cost to the province every year. We expect this information to prove valuable for public health agencies and policy-makers, as it seems advisable to reinstate appropriate surveillance and monitoring systems and to implement effective control measures that avoid the spread and recrudescence of the disease.

## Author Summary

Historically, cystic echinococcosis (CE) is one of the most important zoonotic diseases in Spain. The initiation of a number of control campaigns in the second half of the 1980s has led to a substantial reduction of the number of CE infections both in humans and livestock species. As a consequence of this initial success, obligation to report human CE cases was revoked in 1997 in a number of Spanish autonomous regions that were consequently considered non-endemic for CE. This policy has not been translated to livestock species, where the identification of hydatid cysts and the reporting of prevalence data remain compulsory in all national slaughterhouses. We present here an estimation of the human and animal CE associated monetary losses in the province of Álava, North Spain, for the year 2005. Obtained economic data corroborate the epidemiological findings and demonstrate that CE has important socio-economic consequences in Álava. Taking together, our data suggest that surveillance status of CE in Álava (and very likely in other Spanish regions currently classified as non-endemic areas) should be reassessed. The situation also indicates that more accurate and effective methods for detecting and reporting the disease at the regional and national level are greatly needed.

## Introduction

Cystic echinococcosis (CE or hydatid disease) caused by the larval stage of the taeniid tapeworm *Echinococcus granulosus* is an important zoonotic disease with a worldwide distribution. Domestic transmission of the infection relies on dogs as definitive hosts and a range of livestock ungulate intermediate hosts, mainly sheep and cattle. The disease represents a serious human and animal health concern, causing important economic losses derived from the costs of medical treatment, morbidity, life impairment and fatalities in human cases and decreased productivity and viscera condemnation in livestock species [1]–[4]. Estimation of the CE monetary burden in humans and livestock quantifies the societal impact of the disease, which can aid policymakers in allocating financial and personnel resources. As such, these investigations should be an integral part of any decision regarding priorities for zoonoses control programs [4].

CE is considered endemic in Spain [5], but this classification varies from region to region. Attempts to effectively control the disease began in 1986 with a pilot program in the Autonomous Region (AR) of Navarre in collaboration with the Spanish Ministry of Health and the Mediterranean Zoonoses Control Centre. This initiative was soon after extended to the ARs of Aragon, Castile-La Mancha, La Rioja and Madrid [6], with some other ARs (Castile-León and Extremadura) developing independent control programs. Because of this concerted effort, there has been a marked reduction in the overall incidence rate of human CE and in the prevalence of animal CE over the past 20 years [7], [8]. However, despite the program's success, CE remains a serious health and economic problem in the North-eastern, Central and Western parts of the country [9]. The overall CE-associated economic losses in Spain were estimated to an average of €149 million (95% credible interval, CI: €22 to €394 million) for the year 2005, with human-associated costs constituting 89.1% of total losses [1]. Reporting of human CE is currently mandatory in nine of the 17 Spanish ARs. The Basque Country, to which Álava belongs, is part of the remaining eight ARs that were classified as non-endemic for CE in 1997 based on the initial success achieved after the implementation of control campaigns. This decision resulted in revoking an obligation to report new human CE cases, and henceforth the unavailability of human CE surveillance data from Álava since 1997.

The province of Álava is bordered by the ARs of Navarre, La Rioja, and Castile-Leon, which are considered among the most prevalent regions for CE in Spain [9]. In addition, the prevalence of infection has been estimated to 0.5% among kennel dogs and 8.0% among sheep dogs sampled in Álava in 1996–1999 and 1997–1998, respectively [10], [11]. Moreover, in a recent survey conducted in abattoirs of the Basque Country, the percentage of bovines presenting hydatid cysts at slaughter varied between 1.5% in 2006 and 4.1% in 2008, with a clear increasing trend [12]. These data combined with the considerably large livestock population of 88,000 sheep, 4,800 goat, 40,000 cattle, and 15,000 pigs make it unlikely for Álava to be truly non-endemic for CE.

The aim of this study was to estimate human and animal CE-associated economic losses in 2005 in the province of Álava. The year 2005 was chosen because it had the most complete and accurate set of epidemiological, clinical and economical parameters required for our analyses. It also allowed direct comparison with previous published data at the national scale [1]. To achieve this aim, the annual incidence rate of human CE between 1991 and 2007 and the prevalence of ovine and bovine CE infection at slaughter between 2000 and 2006 were also estimated.

## Materials and Methods

### Ethical statement

Hospital medical records (HMR) used in this survey to gather clinical and socio-demographic data were anonymized prior to any review or analysis in order to preserve the identity of the affected patients. This study was been approved by the Research Ethics Committee of the Health Institute Carlos III.

### Estimation of the annual incidence rate of human CE in Spain and Álava between 1982 and 2007

The official number of reported CE cases in the province of Álava between 1982 and 1996 and in Spain between 1982 and 2007 were obtained from the national epidemiological surveillance network [13]. Since reporting of CE cases in Álava was discontinued in 1997, we reviewed the HMRs of all patients who were diagnosed with CE in the two main hospitals of the province (Txagorritxu Hospital and Santiago Apóstol Hospital) from 1991 to 2007. In Álava, all patients suspected of having hydatid cysts (e.g. positive serology) are referred to one of these hospitals for confirmation and treatment. The population sizes for 1982 to 2007 in Álava were taken from the Basque Statistics Institute [14]. The annual incidence rate (per 100,000 person-years) was estimated by dividing the total number of cases residing in Álava at the time of diagnosis by the estimated population size for that year, and then multiplying by 100,000. Socio-demographic (age group, gender, place of birth and residence at the moment of diagnosis, urban/rural environment) and clinical (methods of diagnosis, cyst type and location, complications presented, treatment adopted, follow-up, re-operations, re-infection, mortality rate) parameters were retrieved from the HMR of each individual CE case attended in the two hospitals between 1991 and 2007.

### Estimation of the prevalence of CE among sheep and cattle slaughtered in Spain and the Basque Country between 1998 and 2006

The numbers of sheep and cattle found to have CE lesions at slaughter in the Basque country during the period 2000–2006 were provided by the Department of Health of the Basque Country. Equivalent figures at the national level between 1998 and 2006 were obtained from the Spanish reports on Trends and Sources of Zoonoses and Zoonotic Agents in Humans, Animals and Foodstuff submitted to the European Food Safety Authority [15]. Regional and national data do include home slaughtered animals infected with CE since by law, every killed animal destined to human consumption must be examined by a veterinarian.

### Human epidemiological and clinical parameters for the 2005 economical valuation

#### Estimation of the frequency of treatment of diagnosed cases

The frequency of different diagnosis and treatment received by the 13 CE cases diagnosed in 2005 was extracted from the HMR data of the two main hospitals in Álava described above (Table 1). Additional epidemiological parameters were obtained from the literature (Table 2). Eight patients were surgically- or chemotherapeutically treated, whereas five patients were left untreated under a watch-and-see policy. Recidival disease was documented in two of the treated patients: one was further subjected to chemotherapy and the remaining one left untreated. Overall, a single 91-years old female patient died in 2005 as a consequence of the infection.

**Table 1:** Distribution of diagnostic tests and treatments received by diagnosed human CE cases in 2005 (13 cases) and for the period between 1991 and 2007 (154 cases) in the two major hospitals capturing all CE cases in Álava.

	Period	
Type of diagnosis and treatment (%)	2005	1991–2007
**Diagnosis**		
Clinical^a^	7.7	10.5
Serology	23.1	15.7
Chest X-ray	15.4	5.2
CT	76.9	73.2
Ultrasonography	30.8	43.8
MRI	7.7	7.8
No Data	0.0	5.2
**Treatment**		
Chemotherapy	15.4	11.8
Surgery	46.1	54.9
Total exeresis	23.1	20.2
Partial exeresis	7.7	28.1
Not specified	15.3	6.6
None	38.5	41.8
No data	0.0	0.6
**Post-treatment recidives** b	61.5	66.2
**Treatment of recidival** c **cases**	Over 2 patients	Over 21 patients
Chemotherapy	50.0	33.3
Surgery	0.0	57.1
Combined	0.0	4.8
None	50.0	4.8
**Follow up**		
Yes	38.5	47.3
Average (Months)	13.5	37.2
No	61.5	46.7
No data	0.0	6.0
**Mortality due to CE**	7.7	3.9
**Length of hospital stay (Days)**	18.4	10.4

a Diagnosis based on signs and symptoms of the disease.

b Patients with recrudescent disease after surgical and/or chemotherapeutical treatment.

c Treatment provided to those patients that experienced recrudescence of the disease after being chemotherapeutical- or surgically treated.

**Table 2:** Additional epidemiological parameters used to estimate the cost of medical treatment for cystic echinococcosis in Álava, 2005.

Item	Frequency	Distribution	Reference
Duration of follow-up	4.5 months	Uniform (3,6)	[39]
Number of follow-up visits to monitor drug response	7.5	Fixed	[40]
Albendazole treatment dose (per day)	800 mg	Fixed	[39]

#### Estimation of the frequency of undiagnosed cases

Diagnosed CE cases are considered to be only the tip of the iceberg when it comes to estimating the monetary burden of CE, since undiagnosed and asymptomatic cases are believed to suffer productivity losses due to the disease [1]–[4], [16]. Yet, no ultrasound surveys to detect CE have been conducted in Spain. Therefore, we used three different approaches to estimate the number of asymptomatic cases. In a first approach, it was assumed that there were no asymptomatic cases. For the two other approaches, we assumed that the prevalence of undiagnosed cases would be proportional to that estimated in the only two available studies linking CE ultrasound-based prevalences and the incidence rates of clinical cases seen in hospital in the same geographic region namely ultrasound surveys conducted in individuals of all ages from rural Uruguay (prevalence of 1.64%) [17] and children in Turkey (prevalence of 0.24%) [18]. In these two studies(), the average annual clinical incidence rates based on hospital cases were 36.1 and between 2 and 3.4 cases per 100,000 person-years (average of 2.7 cases per 100,000 person-years), for ratios of undiagnosed cases to clinical cases seen in hospitals of 45.4 and 88.9, respectively. When applying these ratios to the incidence rate of clinical CE cases seen in hospitals in the province of Álava in 2005 (305,822 inhabitants; 4.25 cases per 100,000 person-years), we estimated the mean prevalence of undiagnosed or asymptomatic CE cases to be between 0.19% and 0.38%. Hence, in our second approach, we used a uniform distribution between the Uruguay estimate (0.19%) and the Turkey estimate (0.38%), while in our third approach we used a triangular distribution between 0% and the Turkey estimate (0.38%), with a mode at the Uruguay estimate (0.19%), to represent the frequency of undiagnosed cases and its uncertainty. The estimated total number of asymptomatic cases under each approach was assumed to follow the age-gender distribution of cases seen in the two reference hospitals between 1991 and 2006.

#### Estimating the level of productivity lost to diagnosed and undiagnosed CE

Seven out of the thirteen CE cases diagnosed in 2005 required hospitalization for diagnosis or treatment procedures. The length of stay of hospitalisation of each of the seven CE inpatients was extracted from the hospital medical charts and used to estimate the wage lost during hospitalization for CE patients. In addition, all diagnosed (inpatients and outpatients) and estimated undiagnosed CE cases were assumed to lose some percentage of their productive time to CE. Although a lower quality of life has been reported among asymptomatic cases of CE [16], the percentage of productivity loss for these cases is unknown. The only available estimate comes from an assumption used in a study aimed at estimating the monetary burden of CE in Uruguay, where asymptomatic cases were assumed to see a 2% decrease in their annual productivity [19]. Due to the very uncertain nature of this estimate, we modelled productivity losses using two distributions: a uniform distribution ranging from 0% to 4% and a general beta distribution with parameters 1 and 3 (average of 1%), and truncated between 0% and 4%. A third approach, using a triangular distribution with minimum and mode values of 0% and a maximum of 4% was explored but resulted in estimates within the range of the other two methods and are therefore not presented. No losses associated with death were included in the analysis since the only death occurring in 2005 was a female whose age was over the life expectancy for Spanish women.

### Source of health provider and non-health provider costs

To estimate the annual overall cost of human CE we included both health provider (direct) costs and non-health provider (indirect) costs. The health provider costs were obtained from the Txagorritxu Hospital financial department for the year 2008 and adjusted the values for 2005, accounting for inflation. Since the health system is public in Spain, the costs in all hospitals in Álava are similar. The cost of each diagnostic test, surgical intervention, and medical treatment reported to have been used for the CE cases treated in 2005 was obtained. Health provider cost estimates included separate calculations for surgical and non-surgical patients.

The non-health provider costs only included productivity losses during hospitalisation for inpatients and productivity losses due to the disease itself for all CE cases. No productivity losses were attributed to medical visits since no information was available on the duration of each visit, and that the work or activities lost during the visit could be completed when the patients returned to their work or home (for retired people). Average wages according to sex and age for the Basque Country, including average pensions for retired people, were obtained from the 2005 Wage Distribution Survey of the National Statistics Institute [20]. We assumed equivalent wages for the province of Álava. The itemised cost menu is presented in Table 3.

**Table 3:** Age-gender stratified wages in the Basque Country in 2005 and costs of treatment and diagnostic test to treat CE patients in Álava, 2005.

Economic Parameters	Value	Reference
**Average yearly wage (**€**)**		
**For males, by age group (years)**		
<25	16,508	[20]
25–34	20,872	[20]
35–44	23,343	[20]
45–54	31,100	[20]
≥55	29,482	[20]
**For females, by age group (years)**		
<25	12,309	[20]
25–34	16,873	[20]
35–44	19,253	[20]
45–54	21,570	[20]
≥55	21,138	[20]
**Cost of diagnostic procedures and medical treatment/care (€ per case)**		
Chest X-ray	9.7–11.9	Uniform distribution from Txagorritxu Hospital
CT	98.6	Txagorritxu Hospital
MRI	222.7	Txagorritxu Hospital
Ultrasonography	38.5	Txagorritxu Hospital
ECG	6.3	Txagorritxu Hospital
Standard analyses	13.1	Txagorritxu Hospital
Serology (hydatidosis)	20.1	Txagorritxu Hospital
Chemotherapy (Mbz/Albz)	59.3	Txagorritxu Hospital
Initial Outpatient medical visit	103.2	Txagorritxu Hospital
Follow-up outpatient medical visits	52.03	Txagorritxu Hospital
CBC count	13.1	Txagorritxu Hospital
**Cost of surgical procedures (€ per case)** a		
	2,832–3,776€	Uniform distribution from Txagorritxu Hospital

a This figure includes the cost of diagnosis, anesthesia, treatment of complications of surgery, post-operation follow-up, re-operations, medical care during hospitalization and hospitalization).

CT, computed tomography;

ECG; electrocardiogram.

Mbz/Albz, mebendazole/albendazole;

MRI, magnetic resonance imaging.

### Livestock epidemiological parameters

Production animal species considered in the analyses included sheep and cattle. Goats and pigs were not considered because none were identified with CE-infection during the study period. Due to the lack of active abattoirs in Álava in 2005, most of the animals from this province were slaughtered somewhere else in the AR of the Basque Country. Therefore, CE infection prevalence proportions in sheep and cattle in Álava were assumed to be the same as those globally reported for the AR of the Basque Country. Final prevalence estimates excluded data from non-autochthonous CE cases (91.1% of infected sheep and 79.2% of cattle were raised and slaughtered in the AR of the Basque Country) [9]. The number of sheep and cattle infected in Álava was estimated by multiplying the prevalence of CE among autochthonous animals slaughtered in the AR of the Basque country by the total population of each species in Álava. Estimates for autochthonous livestock life expectancy and reproductive rates were provided by Carlos Marín Ruiz (Department of Livestock, Regional Government of Álava). Official figures for annual livestock meat and milk production were obtained from the Spanish Ministry of Agriculture, Food and Environment [21]. Data stratified by age (young lambs: <1.5 months; lambs: 1.5 months to 1 year; adult sheep: >1 year old; calves: <1 year old; young cattle: >1 and <2 year old; adult cattle: >2 year old)) were included when available. Productivity losses associated with CE – including reduction in growth, reduction in milk production and decrease in fecundity – were estimated from the published literature available [7], [22]–[25]. All productivity losses estimates were attributed a uniform distribution to reflect their uncertainty (Table 4). The costs of fecundity and growth losses were only estimated for meat animals up to 1 year for sheep (lamb) and 2 years for cattle (beef cattle). The animal epidemiological parameters used in this study are reported in Table 4.

**Table 4:** Epidemiological parameters used to estimate the economic losses associated with CE in livestock, Álava, 2005.

Parameter	Value	Distribution	Range	Unit	Reference
Total sheep population^a^	88,369	Fixed	NA	Individuals	[27]
Lambs^a^	4,600	Fixed	NA	Individuals	[27]
Lambs for slaughter	47,709	Fixed	NA	Individuals	[27]
Proportion of slaughtered lambs aged <1.5 months	28	Fixed	NA	% of slaughtered lambs	[27]
Adults for slaughter	53	Fixed	NA	Individuals	[27]
Ewes producing milk^a^	28,691	Fixed	NA	Individuals	[27]
Ewes producing meat lamb^a^	29,019	Fixed	NA	Individuals	[27]
Adult nulliparous ewes^a^	23,871	Fixed	NA	Individuals	[27]
Males for reproduction	2,187	Fixed	NA	Individuals	[27]
Prevalence of infection at inspection	1.76	Fixed	NA	% of infected animals at slaughter	Calculated
Lambs	1.74	Fixed	NA	% of infected animals at slaughter	Basque Government
Adults	1.79	Fixed	NA	% of infected animals at slaughter	Basque Government
Proportion of infected sheep with lung cysts only	10	Uniform	5–10	% infected sheep at slaughter	[26]
Proportion of infected sheep with liver cysts only	30	Uniform	25–35	% infected sheep at slaughter	[26]
Proportion of infected sheep with lung and liver cysts	60	Uniform	50–70	% infected sheep at slaughter	[26]
Average weight					
Live lamb (<1.5 months)	11	Uniform	8–14	Kg	[20]
Live lamb (1.5 months-1 year)	24	Uniform	22–26	Kg	[20]
Lamb liver	0.85	Uniform	0.8–0.9	Kg	Extrapolated from [41], [42]
Lamb lung	0.60	Uniform	0.5–0.7	Kg	Extrapolated from [41], [42]
Sheep liver	1.00	Uniform	0.9–1.1	Kg	Extrapolated from [43]
Sheep lung	0.70	Uniform	0.6–0.8	Kg	Extrapolated from [43]
Mean lambing/year – meat sheep	1.5	Fixed	NA	Lambs per ewe per year	See text
Average milk yield of dairy sheep	165	Uniform	160–170	Kg per year	Extrapolated from [20]
Total cattle population^a^	40,196	Fixed	NA	Individuals	[27]
Calves (<1 yr. old) for slaughter	170	Fixed	NA	Individuals	[27]
Calves for milk replacement	1,923	Fixed	NA	Individuals	Estimate
Calves for beef cattle	5,869	Fixed	NA	Individuals	Estimate
Calves for beef cattle reproduction	2,096	Fixed	NA	Individuals	Estimate
Beef cattle (>1 and <2 yr. old) for slaughter	3,987	Fixed	NA	Individuals	[27]
Young animal for milk replacement	441	Fixed	NA	Individuals	Estimate
Young animals for beef cattle reproduction	236	Fixed	NA	Individuals	Estimate
Adults (>2 yr. old)a	25,382	Fixed	NA	Individuals	[27]
For slaughtering (cows)	2,393	Fixed	NA	Individuals	[27]
For slaughtering (bulls)	4,665	Fixed	NA	Individuals	[27]
For milk production	6,915	Fixed	NA	Individuals	[27]
No. of cattle slaughtered/year	11,215	Fixed	NA	Individuals	[27]
Calves	170	Fixed	NA	Individuals	[27]
Beef cattle	3987	Fixed	NA	Individuals	[27]
Adult cows	2393	Fixed	NA	Individuals	[27]
Bulls	4665	Fixed	NA	Individuals	[27]
No. of infected cattle slaughtered/year	304	Fixed	NA	Individuals	Calculated
Prevalence of infection at inspection	2.71	Fixed		% of infected animals at slaughter	Basque Government
Proportion of infected cattle with lung lesions only	17.9	Fixed	NA	Percent	[26]
Proportion of infected cattle with liver lesions only	6.4	Fixed	NA	Percent	[26]
Proportion of infected cattle with lung and liver lesions	73.5	Fixed	NA	Percent	[26]
Average weight					
Live calf	160.0	Uniform	130–190	Kg	[20]
Live young cattle	480.00	Uniform	460–500	Kg	[20]
Calf liver	3.20	Uniform	2.9–3.5	Kg	[44]–[46]
Calf lung	3.75	Uniform	3.5–4.0	Kg	[44]–[46]
Young animal lung	5.09				Estimated from calf and cow
Young animal liver	5.25				Estimated from calf and cow
Cow liver	6.35	Uniform	5.4–7.3	Kg	[44]–[46]
Cow lung	6.15	Uniform	5.2–7.1	Kg	[44]–[46]
Mean calving/year – beef cow	1.05	Fixed	NA	Calves per cow per year	See text
Average milk yield of dairy cow	7,064	Fixed	NA	Kg per year	[27]
Productivity losses – all livestock					
Decrease in fecundity	5.5	Uniform	0.0–11.0	% decrease per year	[24]
Decrease in carcass weight	6.25	Uniform	2.5–10.0	% decrease per year	[7], [25]
Decrease in milk production	2.5	Uniform	0.0–5.0	% decrease per year	[7], [25]

a Census at 31 December 2005.

NA, not applicable.

### Estimation of livestock costs

Direct costs (loss of revenue through offal condemnation) and indirect costs (reductions in growth, fecundity, and milk production) were the parameters we used to estimate the overall losses associated with bovine and ovine CE in Álava province in 2005. The itemised cost menu for animal losses is presented in Table 5.

**Table 5:** Cost parameters used to estimate the economic losses associated with CE in sheep and cattle, Álava, 2005.

Parameter	Value	Reference
**Sheep**		
Average value		
Amount paid to farmer for young lamb live carcass (<1.5 months) (€ per 100 kg)	404	[27]
Amount paid to farmer for lamb live carcass (>1.5 months) (€ per 100 kg)	265	
Sheep liver (€ per kg)	0.65	[27]
Sheep lung (€ per kg)	0.09	[27]
Sheep's milk at farm gate (€ per 100 L)	79.11	[21]
Farmer investment (€ per kg)	1.59	Extrapolated from [28]
**Cattle**		
Average value		
Amount paid to farmer for young calf live carcass (<1 year) (€ per 100 kg)	379.95	[27]
Amount paid to farmer for beef cattle live carcass (1–2 year) (€ per 100 kg)	335.09	[27]
Young live cow (€ per kg)	1.76	[21]
Beef carcass (€ per kg)	3.35	Extrapolated from [27]
Cow liver (€ per kg)	0.85	[27]
Cow lung (€ per kg)	0.06	[27]
Cow's milk at farm gate (€ per 100 L)	31.25	[21]
Farmer investment/kg (€ per kg)	2.04	Extrapolated from [28]

#### Offal condemnation (direct costs)

In the province of Álava, like the rest of Spain, identification of hydatid cysts at meat inspection leads to condemnation of infected offal. The distribution of liver, lung, and liver and lung lesions at slaughter was estimated at 5%–15%, 25%–35% and 50%–70% for sheep and 17.9%, 6.4% and 73.5% for cattle, respectively, based on a study conducted in abattoirs in Romania [26]. The direct lost revenue for each CE-infected animal was estimated according to the average weight (see ref. [1]) and market value [27] of both liver and lung by species and age at slaughter. We calculated the direct costs by multiplying the lost earnings at abattoir per animal with the number of reported infected animals in each species group.

#### Growth reduction

A reduced carcass weight of CE infected animals at slaughter was assumed. We first estimated the difference in income from the sale of a healthy carcass and a CE-infected carcass (which will weigh less due to growth reduction) for each species. We calculated the net loss to the farmer per infected animal as the difference between the potential income and the reduced price of an unhealthy animal, accounting for the annual farmer investment for an individual sheep or cow projected to 2005 values [28]. We assumed that farmers would invest equally in a CE-infected or uninfected animals, but the CE infected animal would weigh less. For all species, the annual cost for CE-associated growth reduction was calculated from the product of the loss in net profit per infected animal and the number of infected animals identified at slaughter for each species.

#### Milk production

Estimates of total annual milk production and current CE prevalence in dairy species were used to estimate losses due to reduction in milk production. Based on published estimates of the average reduction in an infected animal's annual milk yield that is attributable to CE, we calculated the potential milk not produced due to infection in each species population. Total costs were calculated by multiplying annual loss of milk in an infected animal by the 2005 dairy market prices for each species.

#### Decreased fecundity

Losses associated with unborn animals were limited to meat producing breeds in sheep and cattle. To simulate the birth rate in the absence of infection, we divided the total number of meat animals born per year by the sum of the number of not infected meat reproductive females and the number of infected meat reproductive females times the reduction in fecundity [24]. The number of meat animal born that would be born in the absence of infection was estimated by multiply the total number of reproductive meat females by the birth rate in the absence of infection. We estimated the total number of unborn animals by calculating the difference between the potential and actual births for each species.

### Sensitivity analyses

We generated five different scenarios to assess the impact of undiagnosed or asymptomatic CE cases in the province of Álava and the annual productivity losses due to CE infection in humans given the variability of both parameters in the available literature [17], [18] (Table 6). Scenario 1 includes wages lost during hospitalization for inpatients but excludes productivity losses and asymptomatic cases. Scenario 2 includes annual productivity losses (uniform distribution between 0% and 4% per year) and applied to both diagnosed cases and estimated number of asymptomatic cases based on a uniform distribution between the Turkey and Uruguay estimates [18]. Scenario 3 uses the same method except that a triangular distribution is applied to estimate the number of asymptomatic cases centred around the Uruguay estimate [17], with minimum prevalence set to zero and using the upper limit of the Turkey estimate as a maximum value [18]. Scenarios 4 and 5 use the same models to estimate the number of asymptomatic cases, but using a Beta distribution ascribed to the reduction in annual productivity.

**Table 6:** Sensitivity analyses and conditions used to estimate the economic losses associated with CE in humans and livestock (sheep and cattle), Álava, 2005.

Scenario	Distribution of the proportion of undiagnosed cases	Distribution of productivity losses percent
1	NA	NA
2	Uniform(0.19,0.38%)^a^	Uniform (0,4%)
3	Triangular(0%,0.19%,0.38%)^a^	Uniform (0,4%)
4	Uniform(0.19%,0.38%)^a^	Beta General (1,3,0,0.04)
5	Triangular(0%,0.19%,0.38%)^a^	Beta General (1,3,0,0.04)

a Extrapolated from data obtained from reference [18] and [19], see text.

NA, not applicable.

For each scenario, 10,000 iterations were generated using Latin hypercube random sampling of the input parameter values and based on their assigned distributions. The 50th percentile of this distribution represents the median, and the 2.5th and 97.5th percentiles represent the 95% credible intervals (CIs) for the total cost of CE per year.

A stepwise linear regression of the estimated costs against the input parameter values (i.e. the parameters with a distribution) was performed to assess the impact of each uncertain parameter on the overall cost estimate. The estimates from models with and without asymptomatic cases and the resulting figures illustrating the impact of input parameters were generated using @Risk Version 5.5.0 software (Palisades Corporation, Ithaca, New York, USA), running as an add-in to Microsoft Excel.

## Results

### Annual incidence rate of human cystic echinococcosis between 1982 and 2007 in Spain and Álava

**Figure 1:**
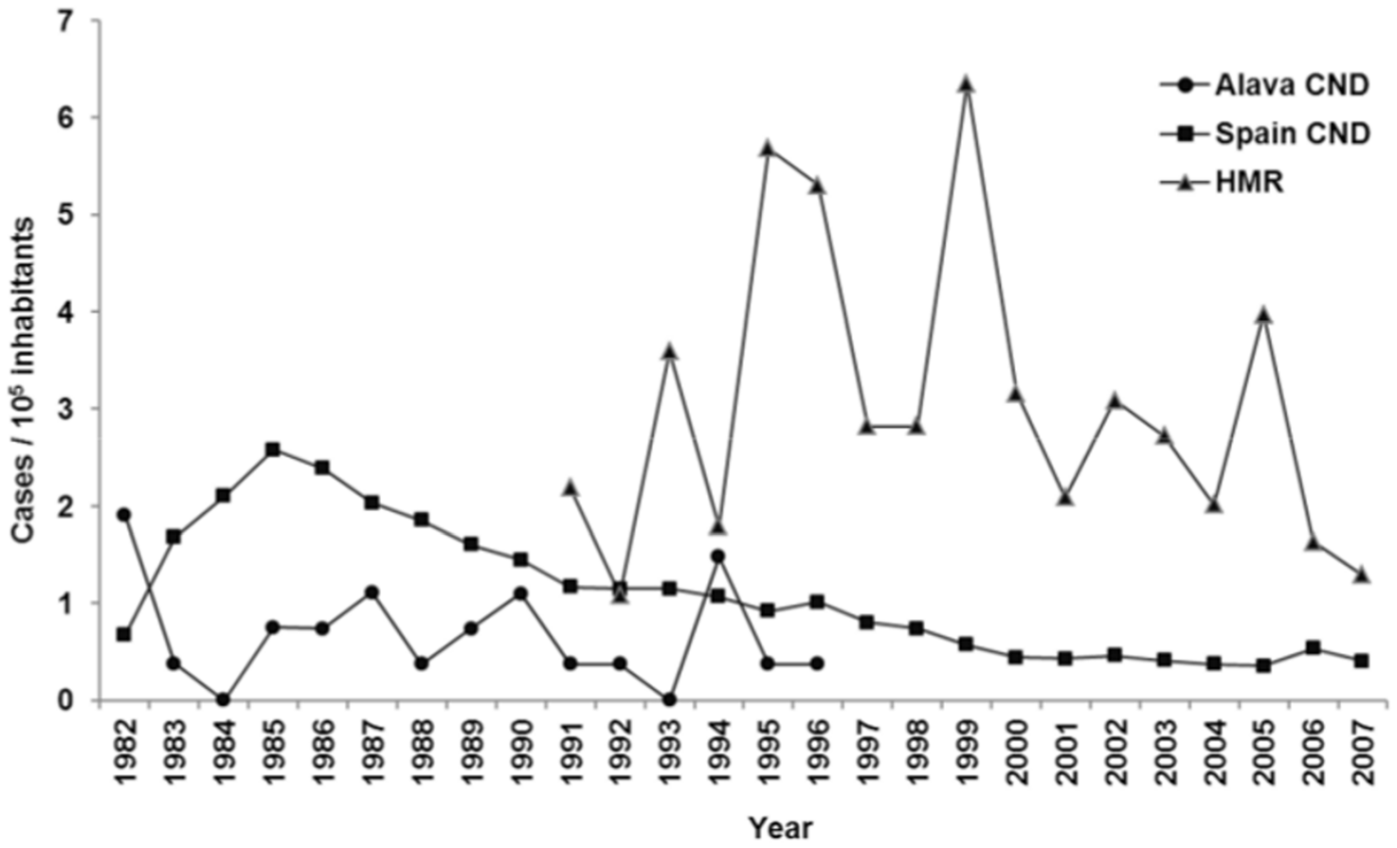
Annual incidence rate per 100,000 inhabitants of cystic echinococcosis in the province of Álava using hospital medical records (1991–2007), and the national surveillance system at the provincial (1982–1996) and national (1982–2007) levels. Legend: Full circles: Estimated annual incidence rate using the Álava Compulsory Notifiable Diseases system; Full squares: Estimated annual incidence rate using the National (Spain) Compulsory Notifiable Diseases system including data from all 17 Spanish autonomous regions (1982–1996) and the nine Spanish autonomous regions considered endemic after (1997–2007); Full triangles: Estimated annual incidence rate using the Hospital Medical Records from the two treating hospitals for CE in Álava. CND: Compulsory Notifiable Diseases system; HMR: Hospital Medical Records.

Figure 1 shows available historical annual incidence rate of human CE in the province of Álava obtained from different sources, including revised HMR (1991–2007) and the official incidence of the disease (1991–1996) provided by the Compulsory Notifiable Diseases system (CND) of the national epidemiological surveillance network [13]. National official incidence rates (1982–2007) have also been included for reference [13]. Not surprisingly, HMR evidenced remarkably higher human CE rates than those reported by the CND, with an average 6-fold increase for the common period between 1991 and 1996. Similarly, a 4-fold increase was observed when the HMR figures were compared to the national official incidence rated for the period between 1997 and 2007.

### Socio-demographic characteristics of human CE cases treated in Álava between 1991 and 2007

**Figure 2:**
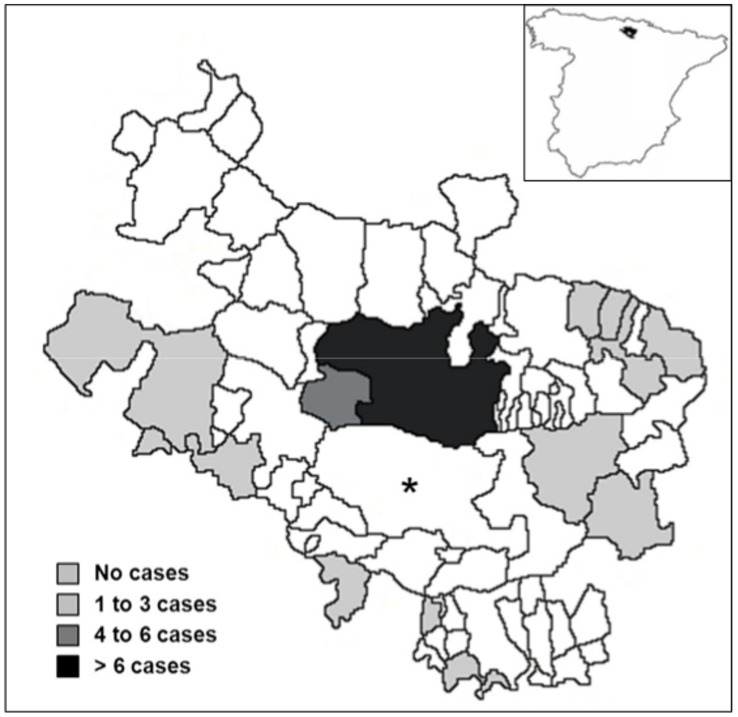
Geographical distribution by municipalities of human CE cases (n = 144) residing in the province of Álava at the moment of diagnosis, 1991–2007. The region pointed out by the asterisk (Treviño) belongs to the adjacent Autonomous Region of Castile-Leon. The geographical location of the province of Álava in Spain is displayed in the top right corner of the figure.

One hundred and fifty-four (154) patients were diagnosed with human CE during 1991–2007 in the two main hospitals of the province of Álava (Table 7). The male/female ratio was 1.05 and the age of cases ranged from 13 to 91 years (mean: 61.5; SD: 17.8). Only three subjects (1.9%) were of paediatric age (defined as ages from birth to 15 years of age), while patients over 60 years old accounted for 61.7% of cases. Most (57.8%) diagnosed cases were native to Álava. Only 4 individuals (2.6%) were born outside of Spain. The vast majority (144 cases, or 93.5%) of cases resided in the province at the moment of diagnosis, with 81.2% living in urban settlements in or around the capital Vitoria-Gasteiz. CE patients residing in rural areas were exclusively distributed alongside the eastern, southern, and western boundaries of the province bordering the ARs of Navarre, La Rioja, and Castile-León, respectively (Figure 2).

**Table 7:** Human CE epidemiological parameters based on individual hospital clinical histories in Álava, 1991–2007.

	2005	1991–2007
Parameter	Value	Value
**Total no. of diagnosed cases**	13	154
In males, by age group, in years	0	1
0–19	0	2
20–29	0	6
30–39	0	8
40–49	2	12
50–59	2	18
60–69	2	21
70–79	0	8
≥80		
In females, by age group, in years	0	3
0–19	0	3
20–29	1	3
30–39	0	10
40–49	1	8
50–59	1	12
60–69	3	20
70–79	1	13
≥80		
No data	0	6
**Gender (%)**		
Male	46.2	51.3
Female	53.8	48.7
No data	0.0	0.7
**Place of birth (%)**		
Álava	53.8	57.8
Rest of the Basque Country	0.0	3.2
Rest of Spain	30.8	31.8
Abroad	0.0	2.6
No data	15.4	4.5
**Place of residence at diagnosis (%)**		
Álava	100	93.5
Rest of the Basque Country	0	2.0
Rest of Spain	0	3.9
Abroad	0	0.0
No data	0	0.6
**Environment (%)**		
Rural	30.8	18.8
Urban	69.2	81.2

Distributions have been indicated for those parameters used to estimate the human economic losses associated with CE in Álava, 2005.

### Comparison of the characteristics of the CE cases diagnosed in Álava in 2005 and between 1991 and 2007

Economic losses for CE in humans in 2005 were based on the 13 cases diagnosed at Txagorritxu Hospital (7 cases) and the Santiago Apóstol Hospital (6 cases). In order to demonstrate the representativeness of the data obtained from the HMR of these 13 patients, we carried out a direct comparison of the average figures of different clinical and epidemiological parameters in 2005 and those corresponding for the whole period 1991–2007. Compared variables included the type of method used to reach a diagnosis, the specific treatment (if any) followed, and also relevant socio-demographic parameters. As clearly shown in Table 1, Table 2, and Table 7, average figures obtained for the years 2005 are in close agreement with those for 1991–2007, demonstrating that the set of data we have used in our analyses did not have unexpected or out of range values and providing evidence of the robustness and accuracy of our results.

### Prevalence of CE among sheep and cattle slaughtered in Spain and Álava between 1998 and 2005

CE prevalence in livestock species (sheep and cattle) for the period 1998–2006 in Spain and the Autonomous Region of the Basque Country were obtained from official sources. Both epidemiological series showed a sustained, declining trend with average ovine and bovine prevalence 2- and 4-fold higher in the Basque Country than in Spain, respectively (data not shown). In the AR of the Basque Country ovine and bovine infections were 2.4% and 6.1% in 2000 and 0.1% and 1.7% in 2006, respectively.

### Economic losses in human and livestock cystic echinococcosis

**Figure 3:**
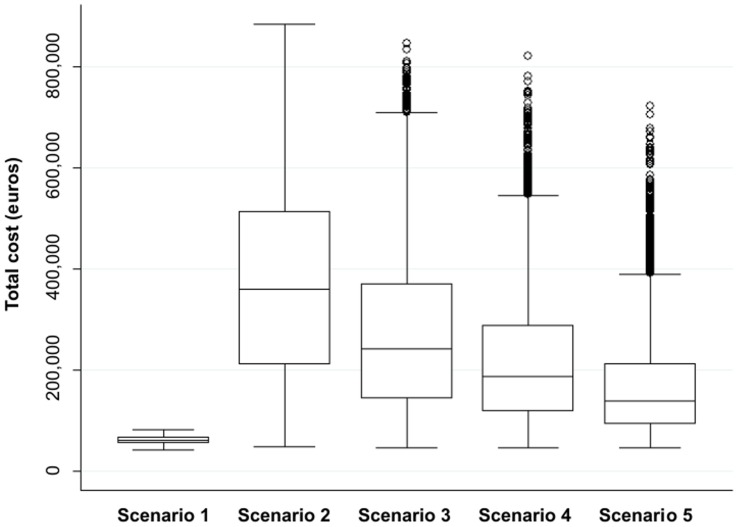
Boxplot representing the estimated total costs of CE in Spain in 2005 under the five scenarios. Legend: Lower line of the box represents the 25th percentile value. Middle line of the box represents the median. High line of the box represents 1.5 the 75th percentile value. The upper whisker represents the 75^th^ percentile plus 1.5 the interquartile range. The lower whisker represents the 25^th^ percentile minus 1.5 the interquartile range. The dots over the high lines represent values that lie outside the lower and upper whisker values (outliers).

The five scenarios run to estimate the total cost of CE in Alava for 2005 showed considerable variation, depending on assumptions made on the prevalence of asymptomatic cases and their associated productivity losses (Table 8 and Figure 3). When asymptomatic cases and productivity losses were excluded, the median cost was estimated at €61,864 (95%CI: €47,304–€76,590). All other scenarios had a lower credible interval superior to €61,000, but the lower whiskers (25^th^ percentile minus 1.5 times the interquartile range) were similar among the models, with a value similar to the median under scenario 1 (see Figure 3). Scenarios 2 and 3, where productivity losses were assumed to follow a uniform distribution between 0% and 4%, resulted in the largest and most uncertain estimates.

**Table 8:** Median costs, in euros, associated with CE in humans and livestock (sheep and cattle), including and excluding asymptomatic or undiagnosed productivity losses in each scenario considered, Álava, 2005.

	Scenario									
	1		2		3		4		5	
	Median	95% CI	Median	95% CI	Median	95% CI	Median	95% CI	Median	95% CI
**Human**										
Direct	27,400	24,681–30,134	27,400	24,681–30,134	27,400	24,681–30,134	27,400	24,681–30,134	27,400	24,681–30,134
Wage loss during hospitalization	8,175	7,857–8,490	8,175	7,857–8,490	8,175	7,857–8,490	8,175	7,857–8,490	8,175	7,857–8,490
Asymptomatic	NI	NI	308,502	22,825–698,762	186,709	15,883–572,971	133,230	13, 301–467,203	83,738	11,014–366,724
Subtotal	35,590	32,813–38,373	335,380	50,460–726,147	214,305	43,449–600,334	160,187	40,633–495,260	111,346	38,368–394,626
**Animal**										
Direct	1,790	1607–1975	1,790	1607–1975	1,790	1607–1975	1,790	1607–1975	1,790	1607–1975
Indirect	24,502	10,071–38,857	24,502	10,071–38,857	24,502	10,071–38,857	24,502	10,071–38,857	24,502	10,071–38,857
Subtotal	26,271	11,869–40,671	26,271	11,869–40,671	26,271	11,869–40,671	26,271	11,869–40,671	26,271	11,869–40,671
**Total**	61, 864	47,304–76,590	360,466	76,424–752,469	241,616	69,064–626,804	187,481	65,215–522,282	138,398	61,167–421,680

NI, not included.

**Figure 4:**
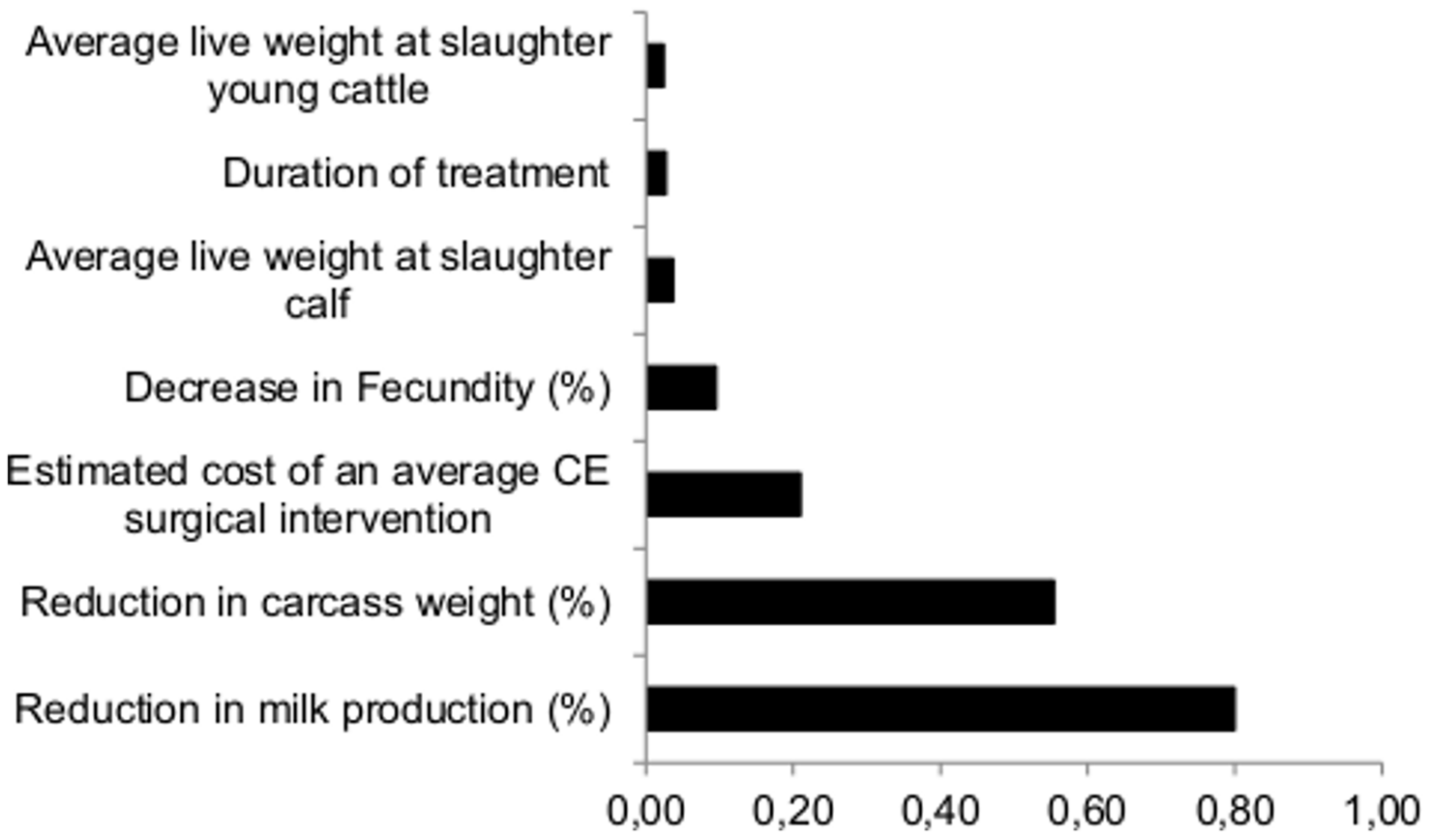
Estimated normalized regression coefficient showing the associations between uncertain parameters and total losses due to CE under scenario 1, Álava, 2005.

Total livestock losses were estimated at €26,425 (95%CI 11,911–40,522), with indirect costs representing 93% of the total costs. Compared to human monetary losses, livestock costs represented 43% of the total costs in scenario 1 whereas they represented only 7% of the total costs in scenario 2, where the total median cost was estimated at €360,466 (95%CI: €76.424–€752,462). Figure 4 shows that most of the variation in the total costs in scenario 1 could be attributed to animal productivity estimates. In scenarios 2–5, the total costs depended largely on the percentage of productivity losses (normalized regression coefficients ranging from 0.82 in scenario 3 to 0.98 in scenario 4), followed by the estimated prevalence of asymptomatic cases in the population (normalized regression coefficients ranging from 0.43 in scenario 5 to 0.53 in scenario 3). The next parameter to contribute to the overall variation was the reduction in milk production with normalized regression coefficients of 0.03 in scenario 2 and 0.06 in scenario 5.

## Discussion

This comprehensive assessment of human and animal CE in Álava demonstrates that CE remains a veterinary public health concern in this Spanish province. Active search of patients diagnosed with CE in HMR demonstrated that the incidence of the disease during the study period is 4- to 6-fold higher than the figures reported in official sources, both at regional and national levels. These results are in-line with recent reports that the CND system underestimates the true incidence of CE disease in Spain [9], [29]–[31]. It should be noted that of the three paediatric patients identified one was born in Mauritania (a 13 years-old male), whereas the other two subjects (two females aged 13 and 14 years, respectively) were born and grew up in the province of Álava. Although acquisition of the infection in other regions/countries cannot be entirely ruled out (e.g. no records of travelling abroad were available), these two patients were almost certainly autochthonous cases. This finding, together with the previous detection of adult *E. granulosus* worms in both urban [10] and rural [11] dog populations, strongly suggests that an active transmission cycle of the parasite is being maintained in Álava. It has been recently reported that between 21–60% of CE cases from Spanish HMR correspond to immigrants from endemic countries [31], [32]. This is not the case in Álava, where only 4 patients (2.6%) were born abroad (one in France, one in Portugal and two in Mauritania). Comparing the proportion of cases among immigrants is difficult, since immigrants in the AR of the Basque Country represent a low percentage of the total population compared to other Spanish ARs.

Regarding livestock CE, the prevalence of the infection in production animal species in the province of Álava was assumed identical to that of the AR of the Basque Country. This assumption is reasonable because i) no operational abattoirs were running in the province during 2005 ii) animals raised in Álava were almost exclusively slaughtered in abattoirs of the AR of the Basque Country, and iii) due to the small size of this AR, the environmental and biological characteristics and the livestock management techniques are homogeneous across the region. The trend of our CE prevalence data series is in agreement with those reported at national level showing slow but sustained reduction in animal infection and ultimately reaching a plateau [9]. This may suggest that CE infection has diminished during the transition from ‘attack phase’ to the ‘consolidation phase’ of the control programs implemented in Spain in the 1980's. Consistent with the human incidence data, average ovine and bovine CE prevalence for the 2000–2004 period were 2- and 4-fold higher in the Basque Country than in Spain, respectively. In this regard, it is worth mentioning that CE bovine infection proportions in the Basque Country have increased from 1.5% in 2006 to 4.6% in 2007 and 4.1% in 2008 [12]. Whether this finding indicates a true re-emergence will require further investigation. Taking together our epidemiological data clearly indicate that both human and animal CE infection rates in the province of Álava/Basque Country are well above the national average figures.

The preferred way to capture both the human and agricultural effect of a zoonosis is to estimate its economic impact [33]. Indeed, to determine the economic burden of human and animal CE is recently identified by international experts as one of the key research priorities in the study of echinococcosis [34]. The aid of computer-based models have been increasingly used in recent years to estimate the socioeconomic burden of CE globally [4] and in a number of endemic regions including Iran [3], Peru [2], Spain [1], the Tibetan plateau [16], [35], and Tunisia [36]. In line with these studies, we present here data on the economic impact of human and animal CE infection in the province of Álava for 2005 based on a Latin Hypercube design. By representing the uncertainty inherently associated to input parameters, this analytical tool is particularly suited for estimating indirect costs where accurate epidemiological information is scarce. Our project design benefits from the incorporation of data at the regional level. For instance, we used the annual CE incidence based on an active search of hospital records rather than the official, potentially underreported figures provided by the national surveillance system. Similarly, CE prevalence proportions in sheep and cattle were obtained from regional abattoir records and appropriately adjusted to exclude non-autochthonous CE infections. In addition, we used stratified rates of human CE infection and average wages by age and gender. For livestock, we used age-stratified prevalence proportions where available.

Our estimates of the monetary burden associated with CE in the province of Álava in 2005 were between €0.06 and €0.36 million. These results reflect the range of variation observed between epidemiological scenarios where productivity losses associated to undiagnosed/asymptomatic infections were excluded, to a worse case situation in which the highest proportion of undiagnosed/asymptomatic CE were considered. All scenarios where asymptomatic cases were included depended largely on the estimated productivity losses and the prevalence of asymptomatic cases in the population. The costs associated with human cases represented from 83% to 94% of the total costs. These results are consistent with our previous estimates for Spain where asymptomatic cases represented 89.1% of the total cost in 2005 [1]. The upper and lower credible intervals of the “worst case scenario” (scenario 2) differed by a factor of ten. This large uncertainty calls for urgent need for more studies of the prevalence of asymptomatic cases in this region and for research on how asymptomatic CE may affect productivity in humans.

The economic burden associated with animal CE was also consistent with our previous estimates for Spain in 2005 [1]. Estimates associated with productivity losses contributed to the largest proportion of the total costs. This demonstrates, as with humans, the urgent need for studies to better estimate productivity losses associated with CE in livestock. In addition, our findings are in line with the fact that *E. granulosus* has been ranked second in the FAO/WHO list of foodborne parasites using a multi-criteria based approach reflecting the number and distribution of global illnesses, morbidity, mortality, the potential for an increased burden, trade relevance, and socio-economic impact [37]. Compared to other zoonotic agents in Spain, our estimated human cost of CE in Álava in 2005 (€35,590–€335,380) is similar to that reported in the province of Málaga, Southwest Spain, for human brucellosis (€347,569/year for the period 1984–1986, projected to 2005 values) [38].

In conclusion, our findings support the idea of an active transmission cycle of *E. granulosus* in the province of Álava primarily maintained between farm dogs acting as definitive host and mainly sheep as intermediate host species. This situation implies that domestic dogs have access to raw viscera of infected animals, most likely by the occasional feeding on carcasses abandoned in the fields. Parasite infection pressure appears sufficient even to induce disease in humans, as demonstrated by the detection of two allegedly autochthonous cases. Documented human and animal CE infection rates are well above national averages, providing strong evidence that the disease is underreported in Spain. Moreover, CE has a significant economic burden in this area, particularly due to indirect costs associated to losses attributed to undiagnosed/asymptomatic cases in humans and reduced productivity in livestock speciesThe results of our analysis also suggest that public health agencies and policy-makers maintain and/or intensify the surveillance and monitoring systems currently in place in order to decrease the prevalence of *E. granulosus* in this area and to avoid the recrudescence of the infection. Ultrasound-based epidemiological surveys in Spanish regions endemic for CE would allow far more accurate estimations on the actual proportions of undiagnosed/asymptomatic human cases. In addition, molecular studies aiming to investigate the frequency of *Echinococcosis* genotypes currently circulating in the province of Álava would be also useful to ascertain the transmission dynamics of the parasite.
